# Prevalence, pattern, and predictors of formal help-seeking for intimate partner violence against women: findings from India’s cross-sectional National Family Health Surveys-3 (2005–2006) and 4 (2015–2016)

**DOI:** 10.1186/s12889-022-14650-3

**Published:** 2022-12-20

**Authors:** Suman Kanougiya, Muthusamy Sivakami, Nayreen Daruwalla, David Osrin

**Affiliations:** 1 School of Health Systems Studies (SHSS), Tata Institute of Social Sciences (TISS), Mumbai, India; 2 Program on Prevention of Violence Against Women and Children, SNEHA, Mumbai, Maharashtra 400017 India; 3 Institute for Global Health, University College London, London, WC1N IEH UK

**Keywords:** Help-seeking, Intimate partner violence, National Family and health surveys, India

## Abstract

**Background:**

Help-seeking for intimate partner violence (IPV) requires women to disclose their experiences. For policymakers, low help-seeking threatens the United Nations Sustainable Development Goals (SDGs) of gender equality, good health, and wellbeing. In India, the Prevention of Domestic Violence Against Women Act (PWDVA 2005) was implemented in 2006. Using two rounds of the India National Family Health Survey (NFHS), one before and one after implementation, we examined the prevalence, pattern, and sociodemographic and socioeconomic factors associated with formal help-seeking for IPV.

**Methods:**

We used univariable and multivariable logistic regression models to assess the prevalence of help-seeking for IPV in the past 12 months and examined associations with different forms of IPV and sociodemographic factors.

**Results:**

The proportion of ever-married women aged 15–49 years who reported physical, sexual, or emotional IPV in the last 12 months increased from 23% in NFHS-3 (2005–2006) to 25% in NFHS-4 (2015–2016). In both surveys, few women sought help. Informal sources of help were preferred over formal sources, which declined from NFHS-3 to NFHS-4 (any help: 24.5 to 13.8%; informal help: 24.1 to 13.4%; and formal help: 1.2 to 1.1%). Women from lower castes and women with children were less likely to seek formal help. Over the two surveys, the odds of formal help-seeking for sexual IPV in the past 12 months remained similar (NFHS-3 aOR 1.9, 95% CI 1.4, 2.5. NFHS-4 aOR 1.9, 95% CI 1.4, 2.6). The odds were slightly higher for emotional IPV (NFHS-3 aOR 2.5, 95% CI 1.8, 3.3. NFHS-4 aOR 2.7, 95% CI 2.0, 3.7) and spousal control (NFHS-3 aOR 2.0, 95% CI 1.4, 3.0. NFHS-4 aOR 2.3, 95% CI: 1.4, 3.7).

**Conclusions:**

Low disclosure and help-seeking impact a country’s social, cultural, economic, and political progress. The PWDVA did not appear to result in increases in help-seeking among women in India who experienced IPV. Further work is needed to understand barriers to help-seeking in the presence of public policy efforts to support women affected by IPV. These may include poor implementation and enforcement of Policy, as well as normalization and justification of gender-based violence. We recommend a deeper understanding of help-seeking across all systems to establish a barometer of help-seeking. An increase in formal or informal help-seeking is an indicator of reduced tolerance of IPV and the enabling environment of the PWDVA 2005 for tracking progress toward the SDGs of gender equality and the eradication of all forms of gender-based violence and discrimination.

## Background

Violations of women’s rights, such as violence against women (VAW), are a priority for the United Nations (UN) Sustainable Development Goals (SDGs) [1]. Male intimate partner violence (IPV) against women is the most common form of VAW [2]. Worldwide in 2018, 27% of ever-partnered women aged 15–49 years were estimated to have experienced physical or sexual IPV worldwide during their lifetime, 13% of them in the past year [3]. Help-seeking by survivors of violence—whether ongoing or in the past—is described as disclosure of violence to obtain some sort of assistance or support [4, 5]. It can be classified into two categories in the context of IPV: (i) formal help from authorities such as health, legal, or police services, shelters, women’s non-governmental organisations (NGOs), or local or religious leaders, and (ii) informal help, which may include but is not limited to, assistance from family, friends, and neighbours [2]. Both formal and informal social support have been found to improve survivors’ mental health, reduce adverse post-traumatic outcomes, reduce the likelihood of future violence, and increase survivors’ willingness and ability to seek formal assistance and subsequent capacity to stay safe [6–10]. For many reasons, however, IPV is globally under-reported [11], and this has limited survivors’ help-seeking [12]. The low proportion of women seeking help for IPV is a major source of concern for policymakers, service providers, and programmes [6].

Help-seeking for IPV is limited in Low and Middle-Income Countries (LMICs) — 40% in Tanzania, Jordan, and Nigeria, 35% in Pakistan, 33% in Bangladesh, 27% in South Sudan, 24% in India, and 20% in Afghanistan and Ethiopia [12–22]. Between 22% and 66% of women who had ever been physically abused told no one about it prior to a World Health Organization (WHO) multi-country survey interview, and 34–59% of survivors of physical violence claimed that no one had attempted to help them, even on request [2]. Informal support was preferred over formal support. Sources of informal support included family (28–63%), friends (18–56%), and neighbours (2–25%); 55–95% of women did not seek help from formal sources [2]. A little over 1% of women survivors of IPV have approached formal sources in India. A relatively large body of work has been carried out, but our collective understanding of the prevalence, pattern, and impact of sociodemographic and socioeconomic factors on formal help-seeking remain understudied.

Several theories or models have been applied to help-seeking behaviour, such as the theory of planned behaviour, Andersen’s behavioural model, and help-seeking and change [10, 23, 24]. Researchers have also identified two internal criteria for help-seeking: survivors perceiving IPV as intolerable and their belief that it is unlikely to resolve without assistance from others [25]. Numerous additional factors influence the decision to seek help and the source of assistance. These include the form of IPV, an increase in severity and frequency of violence, survivors’ fear for their lives, their sociodemographic and socioeconomic status, interpersonal factors, insufficient support systems, and societal norms [2, 26–28].

Another dimension that affects decisional balance for help-seeking is the effectiveness or responsiveness of legislation. In theory at least, legal acts to protect women against domestic violence can help foster an environment that enables survivors to resist, disclose, testify, or seek help [29]. Effectiveness might manifest as, among others, a decrease or increase in reported crimes or an increase in service utilisation, with increased disclosure and access to sources of help [30]. India’s Protection of Women from Domestic Violence Act of 2005 (PWDVA 2005) is the most progressive measure against domestic abuse in Indian history. It is a civil law enacted to address inadequacies in India’s existing domestic violence statutes, specifically Section 498A of 1983 and 304B of 1986 of the Indian Penal Code, which focused on dowry-related assault. The PWDVA 2005 describes physical, sexual, emotional, and economic abuse based on the UN Declaration on VAW [31]. The definition of domestic violence extends to the threat of abuse, including harassment in the form of unlawful dowry demands. It applies to daughters, sisters, widows, mothers, and women in partnerships that resemble marriage and is seen as the first piece of legislation to grant legal recognition and protection for non-marital partnerships. Rather than solely punishing the husband, the PWDVA 2005 is civil legislation intended primarily for the enforcement of protection orders, rather than criminal law. It grants women certain rights: (i) to apply for a protection order, an order for monetary relief, a custody order, a residence order, or a compensation order; (ii) to receive free legal services under the Legal Services Authorities Act of 1987; and (iii) to file a complaint under section 498A of the Indian Penal Code [32]. Through the PWDVA, vulnerable women are also entitled to protection, residence, monetary relief and maintenance, compensation, custody, and legal service.

The PWDVA stipulates that central government and state government officials, including police officers, healthcare providers, and members of the judicial services, must receive “periodic sensitization and awareness training” on the topics addressed by the Act [33]. The Act also authorises the state to issue protective orders (which must be enforced by the police) and to employ Protection Officers to help survivors of domestic violence access medical care and submit domestic abuse reports [33]. Although some newspapers and web commentaries covered the PWDVA, the Act itself did not prescribe how public awareness could be encouraged. Its socialisation among policy implementers, survivors and the public remains an obstacle. National Crime Records Bureau (NCRB) data from 2006 to 2014 suggest that reporting of domestic violence increased in 16 states and remained relatively stable in the other nine [29]. According to an NCRB report, 89,097 cases of crimes against women were filed across India in 2018. The figures indicate that little progress has been made in comparison to the 86,001 instances filed under this heading in 2017. Out of the total crimes against women registered under the Indian Penal Code (IPC), the majority (31.9%) were filed under the ‘cruelty by spouse or his family’ category. At 27.6%, ‘attack against women with the goal to offend her modesty’ came in second. Women’s kidnapping and abduction accounted for 22.5% of all crime, while rape accounted for 10.3% of all crime [34]. The NCRB report compiles crime information from all states. It defines the crime rate as the number of instances reported by the female population in 100,000 s under Section 498A of the IPC. India’s National Family Health Survey (NFHS) is a nationally representative data source that collects self-reported responses about marital violence. NFHS survey rounds are comparable due to compatible designs, including sampling, questionnaire content, and field staff training. We compared two rounds of NFHS data obtained before and after implementing the PWDVA-2005—NHFS-3 (2005–2006) and NFHS-4 (2015–2016)—to see if there was an increase in reported help-seeking from formal resources when women disclosed IPV in the preceding 12 months. We examined (i) the prevalence and pattern of help-seeking by form of IPV, (ii) sociodemographic and socioeconomic factors associated with help-seeking, and (iii) associations between sources of help and forms of IPV.

## Methods

### Setting

The NFHS is a cross-sectional survey that began in 1992–1993. It provides national and state-level estimates to help policymakers and programme administrators plan and administer population, health, and nutrition programmes. The Indian Ministry of Health and Family Welfare started the NFHS surveys with the International Institute for Population Sciences (IIPS) as the nodal agency to produce high-quality data on demographic and health indicators. The datasets include representative urban and rural household samples in all 29 states.

### Design and participants

The NFHS includes a domestic violence module to collect data on coercive control and physical, sexual, and emotional abuse by intimate partners and other family members. The module is administered to a subsample of households selected for the state module. The NFHS uses a stratified two-stage sampling approach in urban and rural areas. The survey report contains a methodology section that includes detailed information on the survey design and data collection [35, 36].

### Sample size

**Fig. 1:**
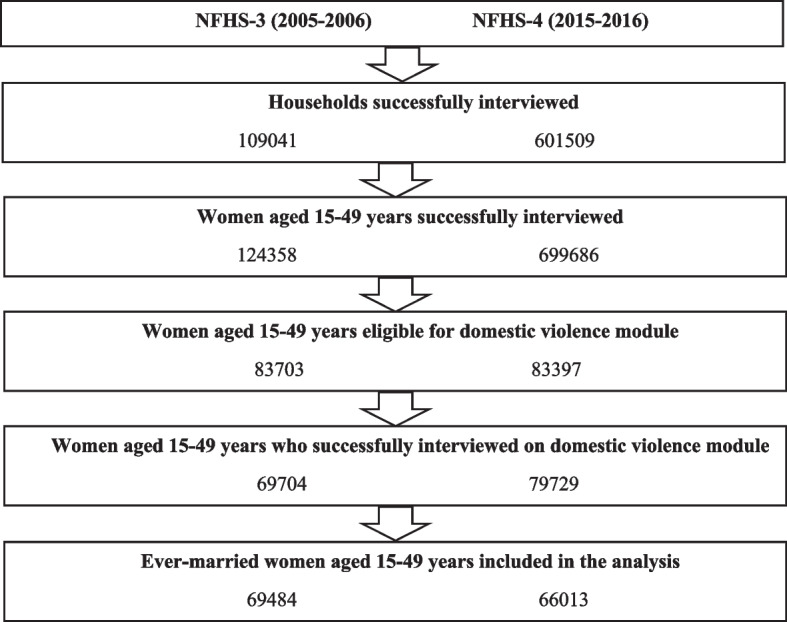
Flow diagram of sampling of ever-married women aged 15–49 years who participated in NFHS-3 & NFHS-4 in India

Figure 1 shows the sampling process. The NFHS-3 of 2005–2006 interviewed 109,041 households (based on the 2001 census enumeration), with a response rate (defined as the number of households interviewed per 100 occupied households) of 98% across India. The domestic violence module was administered to 83,703 women (13,999 never-married women and 69,704 women who had been married) aged 15–49 years [35]. The NFHS-4 of 2015–2016 interviewed 601,509 households (based on the 2011 census enumeration), with a response rate of 98%. The domestic violence module was administered to 79,729 women aged 15–49 years [36]. Women whose marriage had not been confirmed through a *gauna* or other ceremony were omitted from the analysis since they were not asked the domestic violence questions. The study included 69,484 ever-married women aged 15–49 years from the NFHS-3 and 66,013 from the NFHS-4.

### Dependent variable: help-seeking

The NFHS module on domestic violence was based on the modified Conflict Tactics Scale [37] and collected data on the types of IPV experienced by women aged 15–49 years and, additionally, their help-seeking behaviour. The relevant questions were, “*Thinking about what you yourself have experienced among the different things we have been talking about, have you ever tried to seek help to stop the person(s) from doing this to you again? (Yes/No)*, and “*From who have you sought help to stop this?”* Potential help came from both informal and formal sources. The woman’s informal sources comprised her immediate family, her current or former partner or husband, a current or former boyfriend, neighbours, friends, or others. Formal institutions included religious leaders, doctors, non-government organisations, the police, and lawyers. The survey recorded lifetime responses for help-seeking. We analysed help-seeking for IPV in the past 12 months and classified responses into three binary variables describing (i) any help sought, (ii) informal help sought, and (iii) formal help sought.

### Independent variables: intimate partner violence

The NFHS domestic violence module asked questions concerning the respondent’s current husband for women currently married and most recent husband for women formerly but not currently married. IPV was assessed using a set of options following the question, “*Does/did your (last) husband ever do any of the following things to you?”*

Physical IPV: (a) slap you; (b) twist your arm or pull your hair; (c) push you, shake you, or throw something at you; (d) punch you with his fist or with something that could hurt you; (e) kick you, drag you or beat you up; (f) try to choke you or burn you; (g) threaten or attack you with a knife, gun, or any other weapon.

Sexual IPV: (h) physically force you to have sexual intercourse with him even when you did not want to; (i) force you to perform any sexual acts you did not want to.

Emotional IPV: (k) say or do something to humiliate you in front of others; (l) threaten to hurt or harm you or someone close to you; (m) insult you or make you feel bad about yourself.

Women could answer each item *yes* or *no* and, if *yes*, estimate the frequency of the act in the 12 months prior to the survey. A *yes* response to one or more of items (a) to (g) constituted evidence of physical violence, to items (h) or (i) evidence of sexual violence, and to items (k) to (m) evidence of emotional abuse. Responses were coded as a single binary variable for each form of abuse in the 12 months prior to the interview.

Spousal control: spousal control was assessed using the following six binary questions without specifying any time frame. Women were requested to say if the following applied to their relationship with their (last) husband: (a) he is jealous or angry if she talks to other men; (b) he frequently accuses her of being unfaithful; (c) he does not permit her to meet her female friends; (d) he tries to limit her contact with her family; (e) he insists on knowing where she is at all times, and (f) he does not trust her with any money. Women could respond ‘yes’ or ‘no’ to each item. Responses were coded in a single binary variable, where none of the acts was coded as 0 and *yes* to any of the acts was coded as 1. A similar scale was employed in the WHO multi-country study of domestic violence against women [2].

### Sociodemographic and socioeconomic variables

The study included the following sociodemographic and socioeconomic variables based on a socio-ecologic model for IPV [38]: women’s marital status (currently married, widowed, separated, or divorced); age in completed years (categorised as 15–24, 25–34, or 35–49 years); schooling (no education, primary, secondary, or higher); number of living children (0, 1, 2, or 3+), women’s employment (not working or currently working). Spouse characteristics included schooling (no education, primary, secondary, or higher); occupation (not working, non-agricultural, agricultural, skilled or unskilled manual); alcohol use (Yes or No). Household characteristics included urban or rural residence; caste (Scheduled Caste, Scheduled Tribe, Other Backward Caste, or General caste); and religion (Hindu, Muslim, or other); and wealth index corresponding to wealth quintiles (poorest, poorer, middle, richer, and richest). We also included women’s justification of physical violence by their spouses. Women were asked the following five questions: beating is justified if a wife (a) goes out without informing her husband; (b) neglects her children; (c) argues with her husband; (d) refuses to have sex with her husband; or (e) fails to properly cook food. A binary variable was coded 1 if a woman answered yes to any of these five questions.

### Statistical analysis

We analysed NFHS-3 and NFHS-4 datasets independently. First, respondents’ background information was summarised using descriptive statistics. Second, we summarized reporting of forms of IPV and help-seeking behaviour. Third, univariable and multivariable logistic regression models were used to examine sociodemographic and socioeconomic associations with help-seeking for IPV in the past 12 months. Models included the source of help (informal or formal) as outcome variables and sociodemographic and socioeconomic characteristics as exposure variables. Finally, we evaluated the relationships between seeking any help, informal help, or formal help (outcome variables) and forms of IPV (exposure variables) in an adjusted model. For instance, the adjusted models included help-seeking from any source as an outcome variable and past 12 months physical IPV as an exposure variable, after adjusting for the characteristics mentioned above and additional IPV types such as controlling behaviour, sexual, and emotional abuse. Similar analyses were conducted for help-seeking from informal and formal help sources. Analyses were done in STATA 15.0 [39].

### Ethical considerations, enhancing data quality and respondent safety

The United States Centers for Disease Control and Prevention reviewed the research design and content of all NFHS survey questionnaires. The ICF International Inc. Institutional Review Board and the International Institute for Population Sciences (IIPS) Institutional Review Board approved it.

The NFHS implemented the protocol of Lori Heise and Mary Ellsberg [40], and followed the WHO core protocol for the ethical requirements for the multi-country research of women’s health and domestic abuse [41]. Field personnel received additional training in delivering the domestic violence module following the survey’s rapport-building and safety standards, which included coping with crises and emotionally preparing for work. Only one eligible woman per household was selected for the domestic violence module, including an additional informed consent procedure. Respondents were assured of confidentiality and a participant information sheet was distributed in each state and union territory in the appropriate language. It included information about available options and resources for women facing domestic violence and legal assistance and other services, and an address for women in need to obtain information about domestic violence. The participant information sheet was small enough to conceal. The highest priority was to ensure anonymity and if this could not be accomplished the domestic violence module was not administered. Additional information can be found at https://dhsprogram.com/Methodology/Survey-Types/DHS.cfm.

## Results

### Respondent profile

Table 1 summarises the background characteristics of 66,234 ever-married women aged 15–49 years in the NFHS-3 and 66,013 in the NFHS-4. In both surveys, over 95% of women were currently married and around 90% had children. The proportion of women aged 15–24 was higher in NFHS-3 (20%) than in NFHS-4 (16%) and the proportion of women aged 35 to 49 was lower in NFHS-3 (38%) than in NFHS-4 (42%). Rural women’s representation was 56% in the NFHS-3 and 71% in the NFHS-4. The NFHS-3 had more uneducated women (40%) than the NFHS-4 (33%), and the same pattern held for husbands (23% vs 19%). The proportion of working women was 37% in the NFHS-3 and 25% in the NFHS-4. More women in the NFHS-3 (37%) reported their husbands drinking than in the NFHS-4 (32%). Around a third of women were Hindu in both rounds. More women in the NFHS-3 (54%) justified husbands beating their wives than in the NFHS-4 (50%).

**Table 1:** Characteristics of ever-married women aged 15–49 years who completed the domestic violence module in NFHS-3 (2005–2006) and NFHS-4 (2015–2016), India

Demographic Profile	NFHS-3 (2005–2006)		NFHS-4 (2015–2016)	
	n	(%)	n	(%)
Marital Status				
Currently married	65,610	(94.4)	62,716	(95.0)
Widowed, separated, or divorced	3874	(5.6)	3297	(5.0)
Age in completed years				
15–24	13,764	(19.8)	10,489	(15.9)
25–34	29,392	(42.3)	27,568	(41.8)
35–49	26,328	(37.9)	27,956	(42.4)
Number of Living Children				
0	6699	(9.6)	6136	(9.3)
1	12,360	(17.8)	12,610	(19.1)
2	21,184	(30.5)	22,842	(34.6)
3+	29,241	(42.1)	24,425	(37.0)
Education				
No education	27,555	(39.7)	22,028	(33.4)
Primary	10,741	(15.5)	9669	(14.7)
Secondary	25,148	(36.2)	28,187	(42.7)
Higher	6035	(8.7)	6129	(9.3)
Women currently employed	25,606	(36.9)	16,658	(25.2)
Husband’s Education				
No formal education	15,895	(23.1)	12,776	(19.4)
Primary	10,773	(15.6)	9854	(15.0)
Secondary	32,494	(47.2)	34,597	(52.6)
Higher	9700	(14.1)	8579	(13.0)
Husband’s Occupation				
Not employed	1248	(1.8)	2674	(4.1)
Non-agricultural	24,428	(35.3)	20,849	(31.9)
Agricultural	17,581	(25.4)	22,363	(34.3)
Skilled or unskilled manual	26,015	(37.6)	19,399	(29.7)
Husband uses Alcohol or Drug	25,902	(37.3)	20,891	(31.7)
Residence				
Urban	30,522	(43.9)	19,469	(29.5)
Rural	38,962	(56.1)	46,544	(70.5)
Caste				
Scheduled caste	11,970	(18.0)	11,686	(18.6)
Scheduled tribe	9140	(13.7)	12,108	(19.3)
Other Backward Caste	22,139	(33.3)	25,574	(40.7)
General caste	23,308	(35.0)	13,449	(21.4)
Religion				
Hindu	51,660	(74.5)	49,546	(75.1)
Muslim	8597	(12.4)	8614	(13.1)
Other	9103	(13.1)	7814	(11.8)
Household wealth quintile				
Poorest	9734	(14.0)	12,838	(19.5)
Poorer	11,117	(16.0)	13,992	(21.2)
Middle	13,551	(19.5)	13,790	(20.9)
Richer	16,051	(23.1)	13,142	(19.9)
Richest	19,031	(27.4)	12,251	(18.6)
Woman justified spousal violence	35,709	(53.9)	31,991	(49.7)
**All**	**69,484**	**(100.0)**	**66,013**	**(100.0)**

### Prevalence and pattern of IPV and help-seeking for IPV

Table 2 shows the proportions of ever-married women aged 15–49 years in the NFHS-3 and NFHS-4 who reported surviving IPV in their lifetime and in the past 12 months. Between NFHS-3 and NFHS-4, reported lifetime physical, sexual, or emotional IPV declined from 35 to 32%, whereas IPV reported in the past 12 months increased from 23 to 25%. Similarly, reported physical IPV increased from 19 to 22%, emotional IPV from 10 to 11%, and spousal control from 39 to 48%. Reporting of sexual IPV declined from 6 to 5%.

**Table 2:** Proportions of ever-married women aged 15–49 years who reported intimate partner violence (IPV) over the lifetime and in the past 12 months, and who sought support for IPV in the past 12 months. NFHS-3 (2005–2006) and NFHS-4 (2015–2016), India

	NFHS-3 (2005–2006)				NFHS-4 (2015–2016)			
	Lifetime		Past 12 months		Lifetime		Past 12 months	
	n	(%)	n	(%)	n	(%)	n	(%)
Physical, sexual, or emotional IPV	24,502	(35.3)	16,128	(23.2)	20,899	(31.7)	16,589	(25.1)
Physical IPV	21,589	(31.1)	12,879	(18.5)	18,680	(28.3)	14,158	(21.5)
Sexual IPV	5778	(8.3)	4047	(5.8)	3975	(6.0)	3246	(4.9)
Emotional IPV	9814	(14.1)	6879	(9.9)	8372	(12.7)	6944	(10.5)
Spousal control (ongoing)			27,098	(39.0)			31,589	(47.9)
**All**	**69,484**	**(100.0)**	**69,484**	**(100.0)**	**66,013**	**(100.0)**	**66,013**	**(100.0)**
Sought any help	5965	(24.5)	5096	(26.61)	3017	(13.8)	2752	(14.5)
Sought informal help	5865	(24.1)	5008	(26.15)	2924	(13.4)	2664	(14.1)
Sought formal help	294	(1.2)	267	(1.39)	230	(1.1)	215	(1.1)
**All**	**24,384**	**(100.0)**	**19,152**	**(100.0)**	**21,872**	**(100.0)**	**18,957**	**(100.0)**

*NFHS* India National Family Health Survey

The proportion of women who sought help for lifetime IPV declined from NFHS-3 to NFHS-4: any help from 24.5 to 13.8%, informal help from 24.1 to 13.4%, and formal help from 1.2 to 1.1%. The same was true of help-seeking for IPV in the past 12 months: any help from 27 to 15%, informal help from 26 to 14%, and formal help from 1.4 to 1.1%.

Table 3 shows help-seeking for specific forms of IPV in the past 12 months. Between NFHS-3 and NFHS-4, the proportion of women seeking any help for IPV in the past 12 months decreased for physical IPV (28 to 15%), sexual IPV (30 to 23%), emotional IPV (38 to 23%), and spousal control (29 to 16%). Emotional IPV resulted in the greatest proportion of women seeking help. The most favoured source of help was the survivor’s own family (18 to 9%), followed by their husband’s or partner’s family (8 to 5%), neighbours (5 to 1%), and friends (3 to 2%). Among formal sources of help, the police were the most common, followed by religious leaders or non-governmental organisations, lawyers, and doctors. Between NFHS-3 and NFHS-4, the proportion of women seeking help from NGOs declined in absolute frequency (54 to 24), but help from religious leaders increased slightly (52 to 58).

**Table 3:** Proportion of ever-married women aged 15–49 years who sought help for intimate partner violence (IPV) in the past 12 months. NFHS-3 (2005–2006) and NFHS-4 (2015–2016), India

	Any IPV (past 12 months)				Physical IPV (past 12 months)				Sexual IPV (past 12 months)				Emotional IPV (past 12 months)				Spousal Control (ongoing)			
	NFHS-3		NFHS-4		NFHS-3		NFHS-4		NFHS-3		NFHS-4		NFHS-3		NFHS-4		NFHS-3		NFHS-4	
	n	(%)	n	(%)	n	(%)	n	(%)	n	(%)	n	(%)	n	(%)	n	(%)	n	(%)	n	(%)
**Any help (Informal or formal)**	**5096**	**(26.6)**	**2752**	**(14.5)**	**3553**	**(28.0)**	**2073**	**(14.6)**	**1185**	**(29.7)**	**733**	**(22.6)**	**2163**	**(38.1)**	**1331**	**(22.7)**	**3969**	**(29.0)**	**2375**	**(16.2)**
**Any informal help**	**5008**	**(26.2)**	**2664**	**(14.1)**	**3494**	**(27.5)**	**2009**	**(14.2)**	**1153**	**(28.9)**	**702**	**(21.6)**	**2117**	**(37.3)**	**1283**	**(21.9)**	**3896**	**(28.5)**	**2294**	**(15.6)**
Own family	3504	(18.3)	1750	(9.2)	2392	(18.8)	1310	(9.3)	794	(19.9)	471	(14.5)	1502	(26.4)	853	(14.6)	2733	(20.3)	1509	(10.3)
Husband/partner family	1563	(8.2)	926	(4.9)	1138	(9.0)	722	(5.1)	359	(9.0)	254	(7.8)	640	(11.3)	435	(7.4)	1177	(8.7)	791	(5.4)
Current/former partner or husband	43	(0.2)	39	(0.2)	25	(0.2)	29	(0.2)	10	(0.3)	8	(0.3)	14	(0.3)	17	(0.3)	30	(0.2)	29	(0.2)
Current/former boyfriend	5	(0.03)	8	(0.04)	1	(0.0)	7	(0.1)	1	(0.0)	5	(0.2)	1	(0.0)	7	(0.1)	4	(0.0)	8	(0.1)
Neighbor	898	(4.7)	273	(1.4)	681	(5.4)	211	(1.5)	267	(6.7)	81	(2.5)	458	(8.1)	159	(2.7)	680	(5.1)	230	(1.6)
Friends	552	(2.9)	390	(2.1)	404	(3.2)	290	(2.1)	137	(3.4)	99	(3.1)	281	(5.0)	201	(3.4)	420	(3.1)	317	(2.2)
Other	72	(0.4)	43	(0.2)	41	(0.3)	39	(0.3)	25	(0.6)	18	(0.6)	38	(0.7)	25	(0.4)	66	(0.5)	40	(0.3)
**Any formal help**	**267**	**(1.4)**	**215**	**(1.1)**	**175**	**(1.4)**	**160**	**(1.1)**	**89**	**(2.2)**	**77**	**(2.4)**	**142**	**(2.5)**	**132**	**(2.3)**	**231**	**(1.7)**	**196**	**(1.3)**
Religious leader	52	(0.3)	58	(0.3)	41	(0.3)	45	(0.3)	16	(0.4)	18	(0.6)	36	(0.6)	31	(0.5)	44	(0.3)	54	(0.4)
Doctor	19	(0.1)	19	(0.1)	13	(0.1)	16	(0.1)	6	(0.2)	10	(0.3)	8	(0.1)	15	(0.3)	16	(0.1)	19	(0.1)
NGO	54	(0.3)	24	(0.1)	36	(0.3)	15	(0.1)	19	(0.5)	8	(0.3)	29	(0.5)	11	(0.2)	50	(0.4)	22	(0.8)
Police	155	(0.8)	122	(0.6)	98	(0.8)	94	(0.7)	54	(1.4)	48	(1.5)	81	(1.4)	84	(1.4)	132	(1.0)	111	(0.8)
Lawyer	42	(0.2)	31	(0.2)	20	(0.2)	19	(0.1)	10	(0.3)	7	(0.2)	14	(0.3)	17	(0.3)	35	(0.3)	29	(0.2)
**All**	**19,152**	**(100.0)**	**18,957**	**(100.0)**	**12,696**	**(100.0)**	**14,158**	**(100.0)**	**3993**	**(100.0)**	**3246**	**(100.0)**	**5682**	**(100.0**	**5860**	**(100.0)**	**13,681**	**(100.0)**	**14,701**	**(100.0)**

*NFHS* India National Family Health Survey, *NGO* Non-government organisation

### Factors associated with formal help-seeking for IPV

Table 4 summarises the associations in the NFHS-3 and NFHS-4 between help-seeking (any, informal, and formal help) and sociodemographic and socioeconomic factors. Here we summarise the findings of the recent NFHS-4 survey. In the adjusted regression model, the odds of seeking formal help for IPV were greater for widowed, separated, or divorced women, women aged 25 years or older, women with primary or secondary education, working women, and women whose husbands used alcohol. Women with one child and women from OBC and general castes had lower odds of obtaining help from formal sources than women in the reference categories.

**Table 4:** Univariable and multivariable logistic regression models for help-seeking, by source of help for intimate partner violence (IPV) in the past 12 months. NFHS-3 (2005–2006) and NFHS-4 (2015–2016), India

Any help												
	NFHS-3 (2005–2006)						NFHS-4 (2015–2016)					
	OR	[95% CI]	*P*-value	aOR	[95% CI]	*P*-value	OR	[95% CI]	*P*-value	aOR	[95% CI]	*P*-value
**Marital status**												
Currently married	1			1			1			1		
Widowed/Separated/Divorced	2.9	[2.6, 3.2]	0.000	2.6	[2.2, 2.9]	0.000	2.4	[2.1, 2.8]	0.000	2.2	[1.9, 2.5]	0.000
**Age (Years)**												
15–24	1			1			1			1		
25–34	1.1	[1.0, 1.2]	0.003	1.1	[1.0, 1.2]	0.047	1.1	[1.0, 1.2]	0.158	1.0	[0.9, 1.2]	0.607
35–49	1.2	[1.1, 1.3]	0.000	1.1	[1.0, 1.3]	0.047	1.1	[1.0, 1.3]	0.047	1.0	[0.9, 1.2]	0.658
**Number of living children**												
0	1			1			1			1		
1	0.9	[0.8, 1.0]	0.127	0.9	[0.8, 1.0]	0.097	0.8	[0.7, 1.0]	0.012	0.8	[0.7, 1.0]	0.029
2	0.9	[0.8, 1.1]	0.295	0.9	[0.8, 1.1]	0.380	0.8	[0.7, 1.0]	0.012	0.8	[0.7, 1.0]	0.065
3+	0.9	[0.8, 1.0]	0.037	0.9	[0.8, 1.0]	0.053	0.8	[0.7, 0.9]	0.006	0.9	[0.7, 1.0]	0.089
**Education**												
No education	1			1			1			1		
Primary	1.1	[1.0, 1.2]	0.007	1.2	[1.1, 1.3]	0.001	1.1	[0.9, 1.2]	0.310	1.1	[0.9, 1.2]	0.395
Secondary	1.1	[1.0, 1.1]	0.138	1.3	[1.1, 1.4]	0.000	1.0	[1.0, 1.1]	0.343	1.1	[1.0, 1.2]	0.202
Higher	0.9	[0.7, 1.1]	0.246	1.3	[1.0, 1.7]	0.054	1.3	[1.1, 1.5]	0.010	1.3	[1.0, 1.6]	0.050
**Women’s employment**												
Not working	1			1			1			1		
Currently working	1.3	[1.2, 1.4]	0.000	1.1	[1.0, 1.2]	0.003	1.4	[1.3, 1.5]	0.000	1.2	[1.1, 1.4]	0.000
**Husband’s education**												
No education	1			1			1			1		
Primary	1.0	[0.9, 1.0]	0.279	0.9	[0.8, 1.0]	0.177	1.0	[0.9, 1.2]	0.684	1.0	[0.9, 1.2]	0.561
Secondary	0.9	[0.9, 1.0]	0.047	0.9	[0.8, 1.0]	0.018	1.0	[0.9, 1.1]	0.591	1.0	[0.9, 1.2]	0.583
Higher	0.7	[0.6, 0.8]	0.000	0.7	[0.6, 0.8]	0.000	1.1	[0.9, 1.3]	0.261	1.1	[0.9, 1.4]	0.380
**Husband’s occupation**												
Not employed	1			1			1			1		
Non-agricultural	0.7	[0.6, 0.9]	0.009	0.8	[0.6, 1.0]	0.061	1.0	[0.8, 1.2]	0.749	1.0	[0.8, 1.2]	0.667
Agricultural	0.7	[0.6, 0.9]	0.010	0.8	[0.6, 1.0]	0.035	0.9	[0.8, 1.1]	0.473	1.0	[0.8, 1.3]	0.972
Skilled and unskilled manual	0.8	[0.7, 1.0]	0.088	0.8	[0.6, 1.0]	0.071	1.2	[1.0, 1.5]	0.118	1.2	[1.0, 1.6]	0.053
**Husband uses alcohol/drug**												
No	1			1			1			1		
Yes	1.7	[1.6, 1.9]	0.000	1.7	[1.6, 1.8]	0.000	1.7	[1.6, 1.9]	0.000	1.7	[1.6, 1.9]	0.000
**Residence**												
Urban	1			1			1			1		
Rural	1.0	[0.9, 1.1]	0.931	1.1	[1.0, 1.2]	0.014	0.9	[0.9, 1.0]	0.130	1.1	[1.0, 1.2]	0.073
**Caste**												
Scheduled caste	1			1			1			1		
Scheduled tribe	0.8	[0.7, 0.9]	0.000	0.7	[0.6, 0.8]	0.000	0.9	[0.8, 1.0]	0.050	0.8	[0.7, 1.0]	0.015
Other backward caste	1.0	[0.9, 1.0]	0.370	1.0	[0.9, 1.1]	0.981	0.9	[0.8, 1.0]	0.012	0.9	[0.8, 1.0]	0.089
General caste	0.7	[0.7, 08]	0.000	0.7	[0.7, 0.8]	0.000	0.9	[0.8, 1.1]	0.380	0.9	[0.8, 1.1]	0.459
**Religion**												
Hindu	1			1			1			1		
Muslim	0.9	[0.9, 10]	0.005	1.2	[1.1, 1.4]	0.001	0.9	[0.8, 1.1]	0.383	1.1	[0.9, 1.3]	0.265
Other	1.1	[1.1, 12]	0.085	1.1	[1.0, 1.3]	0.038	1.1	[1.0, 1.3]	0.080	1.0	[0.8, 1.2]	0.833
**Wealth quintile**												
Poorest	1			1			1			1		
Poorer	1.1	[1.1, 12]	0.117	1.1	[0.9, 1.2]	0.343	1.0	[0.8, 1.1]	0.386	0.9	[0.8, 1.1]	0.384
Middle	1.1	[1.1, 12]	0.032	1.1	[1.0, 1.2]	0.119	1.0	[0.9, 1.2]	0.522	1.0	[0.9, 1.2]	0.581
Richer	1.0	[1.0, 11]	0.438	1.0	[0.9, 1.2]	0.847	1.1	[0.9, 1.2]	0.258	1.1	[0.9, 1.3]	0.288
Richest	1.0	[1.0, 11]	0.490	1.0	[0.8, 1.2]	0.982	1.2	[1.0, 1.4]	0.009	1.3	[1.1, 1.6]	0.007
**Woman justifies spousal violence**												
No	1			1			1			1		
Yes	0.9	[0.8, 0.9]	0.003	0.9	[0.8, 0.9]	0.001	0.9	[0.8, 1.0]	0.005	0.9	[0.8, 1.0]	0.033
**Informal help**												
	NFHS-3 (2005–2006)						NFHS-4 (2015–2016)					
	OR	[95% CI]	*P*-value	aOR	[95% CI]	*P*-value	OR	[95% CI]	*P*-value	aOR	[95% CI]	*P*-value
**Marital status**												
Currently married	1			1			1			1		
Widowed/Separated/Divorced	2.7	[2.4, 3.1]	0.000	2.4	[2.1, 2.8]	0.000	2.3	[2.0, 2.7]	0.000	2.1	[1.8, 2.5]	0.000
**Age (Years)**												
15–24	1			1			1			1		
25–34	1.1	[1.0, 1.2]	0.004	1.1	[1.0, 1.2]	0.061	1.1	[1.0, 1.2]	0.196	1.0	[0.9, 1.2]	0.899
35–49	1.2	[1.1, 1.3]	0.000	1.1	[1.0, 1.2]	0.096	1.1	[1.0, 1.3]	0.097	1.0	[0.8, 1.2]	0.908
**Number of living children**												
0	1			1			1			1		
1	0.9	[0.8, 1.0]	0.073	0.9	[0.7, 1.0]	0.053	0.8	[0.7, 1.0]	0.020	0.8	[0.7, 1.0]	0.051
2	0.9	[0.8, 1.0]	0.224	0.9	[0.8, 1.1]	0.309	0.8	[0.7, 1.0]	0.022	0.9	[0.7, 1.0]	0.126
3+	0.9	[0.8, 1.0]	0.025	0.9	[0.7, 1.0]	0.036	0.8	[0.7, 1.0]	0.011	0.9	[0.7, 1.0]	0.144
**Education**												
No education	1			1			1			1		
Primary	1.1	[1.0, 1.2]	0.016	1.2	[1.1, 1.3]	0.003	1.0	[0.9, 1.2]	0.558	1.0	[0.9, 1.2]	0.695
Secondary	1.1	[1.0, 1.1]	0.167	1.2	[1.1, 1.4]	0.000	1.0	[0.9, 1.1]	0.710	1.0	[0.9, 1.2]	0.614
Higher	0.9	[0.7, 1.1]	0.286	1.3	[1.0, 1.7]	0.045	1.3	[1.1, 1.5]	0.014	1.3	[1.0, 1.6]	0.059
**Women’s employment**												
Not working	1			1			1			1		
Currently working	1.3	[1.2, 1.3]	0.000	1.1	[1.0, 1.2]	0.004	1.3	[1.2, 1.5]	0.000	1.2	[1.1, 1.4]	0.000
**Husband’s education**												
No education	1			1			1			1		
Primary	1.0	[0.9, 1.0]	0.354	0.9	[0.8, 1.0]	0.232	1.0	[0.9, 1.1]	0.959	1.0	[0.9, 1.2]	0.717
Secondary	0.9	[0.9, 1.0]	0.053	0.9	[0.8, 1.0]	0.021	1.0	[0.9, 1.1]	0.620	1.1	[0.9, 1.2]	0.438
Higher	0.7	[0.6, 0.8]	0.000	0.7	[0.6, 0.8]	0.000	1.1	[0.9, 1.3]	0.330	1.1	[0.9, 1.4]	0.383
**Husband’s occupation**												
Not employed	1			1			1			1		
Non-agricultural	0.7	[0.6, 0.9]	0.006	0.8	[0.6, 1.0]	0.041	1.0	[0.8, 1.2]	0.882	1.0	[0.8, 1.2]	0.833
Agricultural	0.7	[0.6, 0.9]	0.009	0.8	[0.6, 1.0]	0.028	1.0	[0.8, 1.2]	0.721	1.0	[0.8, 1.3]	0.675
Skilled and unskilled manual	0.8	[0.7, 1.0]	0.067	0.8	[0.6, 1.0]	0.052	1.2	[1.0, 1.5]	0.090	1.3	[1.0, 1.6]	0.038
**Husband uses alcohol/drug**												
No	1			1			1			1		
Yes	1.7	[1.6, 1.8]	0.000	1.7	[1.6, 1.8]	0.000	1.7	[1.6, 1.9]	0.000	1.7	[1.6, 1.9]	0.000
**Residence**												
Urban	1			1			1			1		
Rural	1.0	[0.9, 1.1]	0.710	1.1	[1.0, 1.2]	0.011	1.0	[0.9, 1.0]	0.283	1.1	[1.0, 1.3]	0.044
**Caste**												
Scheduled caste	1			1			1			1		
Scheduled tribe	0.8	[0.7, 0.9]	0.000	0.7	[0.6, 0.8]	0.000	0.9	[0.8, 1.0]	0.104	0.8	[0.7, 1.0]	0.022
Other backward caste	1.0	[0.9, 1.1]	0.591	1.0	[0.9, 1.1]	0.723	0.9	[0.8, 1.0]	0.028	0.9	[0.8, 1.0]	0.152
General caste	0.8	[0.7, 0.8]	0.000	0.7	[0.7, 0.8]	0.000	1.0	[0.8, 1.1]	0.655	1.0	[0.8, 1.1]	0.729
**Religion**												
Hindu	1			1			1			1		
Muslim	0.9	[0.8, 1.0]	0.004	1.2	[1.1, 1.4]	0.001	1.0	[0.8, 1.1]	0.588	1.1	[0.9, 1.3]	0.197
Other	1.1	[1.0, 1.2]	0.070	1.1	[1.0, 1.3]	0.038	1.1	[1.0, 1.3]	0.057	1.0	[0.9, 1.2]	0.976
**Wealth quintile**												
Poorest	1			1			1			1		
Poorer	1.1	[1.0, 1.2]	0.136	1.1	[0.9, 1.2]	0.323	0.9	[0.8, 1.0]	0.135	0.9	[0.8, 1.0]	0.168
Middle	1.1	[1.0, 1.2]	0.044	1.1	[1.0, 1.2]	0.095	1.0	[0.9, 1.2]	0.687	1.0	[0.9, 1.2]	0.623
Richer	1.0	[0.9, 1.1]	0.632	1.0	[0.9, 1.2]	0.853	1.1	[0.9, 1.2]	0.313	1.1	[0.9, 1.3]	0.233
Richest	1.0	[0.9, 1.1]	0.511	1.0	[0.9, 1.2]	0.865	1.2	[1.0, 1.4]	0.016	1.3	[1.1, 1.6]	0.007
**Woman justifies spousal violence**												
No	1			1			1			1		
Yes	0.9	[0.8, 1.0]	0.006	0.9	[0.8, 1.0]	0.001	0.9	[0.8, 1.0]	0.006	0.9	[0.8, 1.0]	0.040
**Formal Help**												
	NFHS-3 (2005–2006)						NFHS-4 (2015–2016)					
	OR	[95% CI]	*P*-value	aOR	[95% CI]	*P*-value	OR	[95% CI]	*P*-value	aOR	[95% CI]	*P*-value
**Marital status**												
Currently married	1			1			1			1		
Widowed/Separated/Divorced	6.4	[6.4, 8.3]	0.000	4.9	[3.6, 6.7]	0.000	5.2	[3.8, 7.1]	0.000	3.7	[2.5,5.3]	0.000
**Age (Years)**												
15–24	1			1			1			1		
25–34	1.6	[1.6, 2.3]	0.020	1.6	[1.0, 2.4]	0.046	1.5	[0.9, 2.4]	0.101	2.0	[1.2,3.5]	0.014
35–49	2.2	[2.2, 3.2]	0.000	2.0	[1.3, 3.2]	0.003	1.9	[1.2, 3.0]	0.007	2.5	[1.4,4.5]	0.002
**Number of living children**												
0	1			1			1			1		
1	2.0	[2.0, 3.5]	0.016	2.2	[1.2, 4.2]	0.011	0.5	[0.3, 0.9]	0.021	0.6	[0.3,1.0]	0.040
2	1.5	[1.5, 2.6]	0.118	1.7	[0.9, 3.2]	0.086	0.6	[0.4, 0.9]	0.028	0.6	[0.4,1.0]	0.037
3+	1.1	[1.1, 1.9]	0.695	1.4	[0.8, 2.6]	0.285	0.5	[0.3, 0.8]	0.001	0.5	[0.3,0.9]	0.016
**Education**												
No education	1			1			1			1		
Primary	1.7	[1.7, 2.3]	0.001	1.8	[1.2, 2.5]	0.002	1.6	[1.1, 2.3]	0.021	1.7	[1.1,2.6]	0.025
Secondary	1.6	[1.6, 2.1]	0.003	1.8	[1.2, 2.6]	0.002	1.7	[1.3, 2.4]	0.001	2.4	[1.6,3.6]	0.000
Higher	2.0	[2.0, 3.8]	0.027	2.4	[1.1, 5.4]	0.029	1.5	[0.8, 2.9]	0.182	1.7	[0.7,3.9]	0.233
**Women’s employment**												
Not working	1			1			1			1		
Currently working	1.6	[1.6, 2.1]	0.000	1.2	[0.9, 1.6]	0.148	2.0	[1.5, 2.6]	0.000	1.7	[1.2,2.3]	0.001
**Husband’s education**												
No education	1			1			1			1		
Primary	1.1	[1.1, 1.6]	0.620	1.1	[0.7, 1.6]	0.779	1.5	[1.0, 2.2]	0.051	1.4	[0.9,2.1]	0.140
Secondary	1.2	[1.2, 1.6]	0.211	1.1	[0.7, 1.5]	0.725	1.1	[0.8, 1.5]	0.631	0.9	[0.6,1.4]	0.701
Higher	1.0	[1.0, 1.7]	0.996	0.8	[0.4, 1.5]	0.453	1.0	[0.6, 1.9]	0.882	0.9	[0.4,1.9]	0.824
**Husband’s occupation**												
Not employed	1			1			1			1		
Non-agricultural	0.6	[0.6, 1.2]	0.175	0.7	[0.3, 1.4]	0.287	1.0	[0.5, 2.0]	0.906	0.9	[0.4,1.9]	0.773
Agricultural	0.4	[0.4, 0.8]	0.008	0.5	[0.2, 1.0]	0.056	0.8	[0.4, 1.7]	0.586	0.9	[0.4,1.8]	0.669
Skilled and unskilled manual	0.6	[0.6, 1.1]	0.076	0.5	[0.3, 1.1]	0.080	1.3	[0.7, 2.6]	0.439	1.2	[0.6,2.5]	0.545
**Husband uses alcohol/drug**												
No	1			1			1			1		
Yes	2.1	[2.1, 2.8]	0.000	1.9	[1.4, 2.6]	0.000	2.5	[1.8, 3.3]	0.000	2.3	[1.7,3.2]	0.000
**Residence**												
Urban	1			1			1			1		
Rural	0.7	[0.7, 0.9]	0.008	1.0	[0.8, 1.4]	0.801	0.7	[0.5, 0.9]	0.011	0.9	[0.7,1.3]	0.721
**Caste**												
Scheduled caste	1			1			1			1		
Scheduled tribe	0.4	[0.4, 0.6]	0.000	0.3	[0.2, 0.6]	0.000	0.6	[0.4, 1.0]	0.040	0.7	[0.4,1.1]	0.100
Other backward caste	0.7	[0.7, 1.0]	0.059	0.7	[0.5, 1.0]	0.052	0.7	[0.5, 1.0]	0.055	0.7	[0.5,1.0]	0.080
General caste	0.9	[0.9, 1.2]	0.508	0.7	[0.5, 1.0]	0.090	0.6	[0.4, 1.0]	0.037	0.6	[0.3,0.9]	0.025
**Religion**												
Hindu	1			1			1			1		
Muslim	1.0	[1.0, 1.5]	0.809	1.6	[1.0, 2.4]	0.034	0.9	[0.6, 1.4]	0.587	1.2	[0.7,2.2]	0.488
Other	0.7	[0.7, 1.1]	0.126	0.8	[0.5, 1.4]	0.462	0.9	[0.6, 1.5]	0.732	0.7	[0.4,1.2]	0.237
**Wealth quintile**												
Poorest	1			1			1			1		
Poorer	1.1	[1.1, 1.7]	0.496	0.8	[0.5, 1.3]	0.361	1.3	[0.9, 1.9]	0.159	1.1	[0.7,1.7]	0.659
Middle	1.2	[1.2, 1.8]	0.295	0.8	[0.5, 1.2]	0.235	1.1	[0.8, 1.7]	0.538	0.9	[0.6,1.4]	0.617
Richer	1.4	[1.4, 2.0]	0.107	0.7	[0.4, 1.1]	0.142	1.4	[0.9, 2.2]	0.091	1.1	[0.6,1.8]	0.792
Richest	1.3	[1.3, 2.0]	0.199	0.6	[0.3, 1.1]	0.098	1.5	[0.9, 2.3]	0.114	1.3	[0.7,2.4]	0.426
**Woman justifies spousal violence**												
No	1			1			1			1		
Yes	0.6	[0.6, 0.8]	0.000	0.7	[0.5, 0.9]	0.005	0.8	[0.6, 1.1]	0.115	0.8	[0.6,1.1]	0.133

*NFHS* National Family Health Survey, India, *CI* 95% confidence interval, *OR* Crude odds ratio, *aOR* Adjusted odds ratio with covariates summarized in Table 4

### Formal help-seeking for forms of IPV in the past 12 months

Associations between help-seeking for forms of IPV in the past 12 months were explored in univariable and multivariable logistic regression models. Sources of assistance were modelled as any help compared with no help, informal help compared with no help, and formal help compared with no help., The multivariable model was adjusted for sociodemographic and socioeconomic factors and other forms of IPV.

Table 5 shows the odds of seeking help for IPV in the preceding 12 months. In the NFHS-4, sexual IPV was associated with greater odds of help-seeking (any help aOR2 1.6, 95% CI 1.4, 1.7; informal help 1.5, 1.4, 1.7; formal help 1.9, 1.4, 2.6), followed by emotional IPV (any help 2.1, 1.9, 2.3; informal help 2.0, 1.8, 2.2; formal help 2.7, 2.0–3.7) and spousal control (any help 1.7, 1.5, 1.9; formal help 1.7, 1.5, 1.9; formal help 2.3, 1.4, 3.7). Between NFHS-3 and NFHS-4, the odds of seeking formal help for sexual IPV in the preceding 12 months remained unchanged (1.9 to 1.9), but increased for emotional IPV (2.5 to 2.7) and spousal control (2.0 to 2.3).

**Table 5:** Univariable and multivariable logistic regression models for source of help for intimate partner violence (IPV) in the past 12 months. NFHS-3 (2005–2006) and NFHS-4 (2015–2016), India

	NFHS-3									NFHS-4								
	OR	[95% CI]	*P*-Value	aOR1	[95% CI]	*P*-Value	aOR2	[95% CI]	*P*-Value	OR	[95% CI]	*P*-Value	aOR1	[95% CI]	*P*-Value	aOR2	[95% CI]	*P*-Value
	Any help for the past 12-month intimate partner violence																	
Any IPV in last 12 months																		
No	1			1			1			1			1			1		
Yes	1.4	[1.3, 1.5]	0.000	1.6	[1.5, 1.7]	0.000	1.5	[1.4, 1.6]	0.000	1.3	[1.2, 1.4]	0.000	1.3	[1.2, 1.4]	0.000	1.2	[1.1, 1.3]	0.000
Physical IPV in last 12 months																		
No	1			1			1			1			1			1		
Yes	1.2	[1.2, 1.3]	0.000	1.4	[1.3, 1.6]	0.000	1.3	[1.2, 1.4]	0.000	1.0	[0.9, 1.1]	0.402	1.1	[1.0, 1.2]	0.115	1.0	[1.0, 1.1]	0.420
Sexual IPV in last 12 months																		
No	1			1			1			1			1			1		
Yes	1.2	[1.1, 1.3]	0.000	1.3	[1.2, 1.5]	0.000	1.2	[1.1, 1.3]	0.000	2.0	[1.8, 2.2]	0.000	2.0	[1.8, 2.2]	0.000	1.6	[1.4, 1.7]	0.000
Emotional IPV in last 12 months																		
No	1			1			1			1			1			1		
Yes	2.2	[2.1, 2.4]	0.000	2.3	[2.1, 2.4]	0.000	2.0	[1.9, 2.2]	0.000	2.4	[2.2, 2.6]	0.000	2.3	[2.1, 2.5]	0.000	2.1	[1.9, 2.3]	0.000
Spousal control																		
No	1			1			1			1			1			1		
Yes	1.6	[1.5, 1.7]	0.000	1.5	[1.3, 1.6]	0.000	1.4	[1.3, 1.5]	0.000	2.0	[1.8, 2.2]	0.000	1.9	[1.7, 2.2]	0.000	1.7	[1.5, 1.9]	0.000
	Informal help for the past 12-month intimate partner violence																	
Any IPV in last 12 months																		
No	1			1			1			1			1			1		
Yes	1.4	[1.3, 1.5]	0.000	1.6	[1.5, 1.7]	0.000	1.4	[1.3, 1.6]	0.000	1.2	[1.2, 1.4]	0.000	1.3	[1.2, 1.4]	0.000	1.2	[1.1, 1.3]	0.000
Physical IPV in last 12 months																		
No	1			1			1			1			1			1		
Yes	1.2	[1.2, 1.3]	0.000	1.4	[1.3, 1.6]	0.000	1.3	[1.2, 1.4]	0.000	1.0	[1.0, 1.2]	0.351	1.1	[1.0, 1.2]	0.101	1.0	[0.9, 1.1]	0.484
Sexual IPV in last 12 months																		
No	1			1			1			1			1			1		
Yes	1.2	[1.1, 1.3]	0.000	1.3	[1.2, 1.4]	0.000	1.2	[1.1, 1.3]	0.001	1.9	[1.8, 2.1]	0.000	1.9	[1.7, 2.1]	0.000	1.5	[1.4, 1.7]	0.000
Emotional IPV in last 12 months																		
No	1			1			1			1			1			1		
Yes	2.2	[2.0, 2.3]	0.000	2.2	[2.1, 2.4]	0.000	2.0	[1.8, 2.2]	0.000	2.4	[2.2, 2.6]	0.000	2.3	[2.1, 2.5]	0.000	2.0	[1.8, 2.2]	0.000
Spousal control																		
No	1			1			1			1			1			1		
Yes	1.6	[1.4, 1.7]	0.000	1.4	[1.3, 1.6]	0.000	1.4	[1.3, 1.5]	0.000	1.9	[1.7, 2.2]	0.000	1.9	[1.7, 2.1]	0.000	1.7	[1.5, 1.9]	0.000
	Formal help for the past 12-month intimate partner violence																	
Any IPV in last 12 months																		
No	1			1			1			1			1			1		
Yes	1.2	[0.9, 1.5]	0.246	2.1	[1.5, 2.8]	0.000	1.8	[1.3, 2.4]	0.000	1.3	[1.0, 1.8]	0.082	1.6	[1.1, 2.2]	0.006	1.4	[1.0, 2.1]	0.050
Physical IPV in last 12 months																		
No	1			1			1			1			1			1		
Yes	1.0	[0.7, 1.2]	0.795	1.7	[1.2, 2.3]	0.001	1.2	[0.9, 1.7]	0.267	1.0	[0.7, 1.3]	0.928	1.2	[0.8, 1.6]	0.390	0.8	[0.6, 1.2]	0.327
Sexual IPV in last 12 months																		
No	1			1			1			1			1			1		
Yes	1.9	[1.5, 2.5]	0.000	2.4	[1.8, 3.2]	0.000	1.9	[1.4, 2.5]	0.000	2.7	[2.1, 3.6]	0.000	2.6	[1.9, 3.5]	0.000	1.9	[1.4, 2.6]	0.000
Emotional IPV in last 12 months																		
No	1			1			1			1			1			1		
Yes	2.7	[2.1, 3.5]	0.000	3.1	[2.4, 4.1]	0.000	2.5	[1.8, 3.3]	0.000	3.6	[2.7, 4.8]	0.000	3.1	[2.4, 4.2]	0.000	2.7	[2.0, 3.7]	0.000
Spousal control																		
No	1			1			1			1			1			1		
Yes	2.9	[2.0, 4.1]	0.000	2.2	[1.5, 3.3]	0.000	2.0	[1.4, 3.0]	0.000	3.0	[1.9, 4.8]	0.000	2.8	[1.7, 4.6]	0.000	2.3	[1.4, 3.7]	0.001

*NFHS* National Family Health Survey, India

*Any IPV* Physical, sexual or emotional IPV in last 12 months

*OR* Crude odds ratio

*aOR1* Odds ratio adjusted with covariates for respondent marital status, age, number of children, education, religion, caste, socioeconomic quintile, respondent and husband education, employment, occupation, husband alcohol use, and women justifying spousal violence

*aOR2* Odds ratio adjusted with covariates for women’s marital status, age, number of children, education, religion, caste, socioeconomic quintile, respondent and husband education, employment, occupation, husband alcohol use, and women justifying wife-beating by husband plus covariates for physical, sexual, emotional IPV and spousal control

## Discussion

Between the NFHS-3 and NFHS-4, reported physical, sexual, and emotional IPV in the past 12 months increased among ever-married women aged 15–49 years. In both surveys, a small proportion of women sought any help for any form of IPV. This proportion declined from NFHS-3 to NFHS-4 for both formal and informal sources. More women continued to seek help from informal than from formal sources, and the odds of help-seeking varied with the form of IPV (greatest for emotional or sexual IPV or spousal coercive control) and sociodemographic position. The odds of help-seeking from formal sources remained similar for sexual IPV and increased a little for emotional IPV and spousal control. Age is an important predictor of the experience of violence as well as help-seeking, but a separate analysis of help-seeking by age did not change the findings.

The PWDVA was implemented in 2006, between the two rounds of the NFHS. The Act is a reforming piece of legislation aimed at reducing domestic violence in India and includes specific provisions. The NFHS-4 of 2015–2016 was collected a decade after implementation and one would expect a consequent reduction in reported IPV, or at least an increase in help-seeking from formal sources. However, the proportion of women who reported IPV increased over the decade between surveys and the proportion of women who sought help was similar or perhaps a little less.

We can see three possible explanations for the findings. First, little may have changed in the wake of the Act. Women continued to experience similar or higher levels of IPV and were as reticent to seek help as previously. One of the reasons for low help-seeking may have been lack of awareness of the PWDVA. Second, willingness to disclose IPV may have increased, leading to higher reported prevalence. Third, the actual prevalence of IPV may have declined, compensated for by this higher reporting and similar levels of help-seeking. Another possible reason for declines in help-seeking may be that the PWDVA had the paradoxical effect of increasing women’s fear of negative consequences if they were to seek help. It is possible that women hesitated to seek help for fear of threats to their safety, as the involvement of law enforcement, paired with poor implementation of the protective aspects of the PWDVA, might further strain their relationships with intimate partners. There is a need to better implement protection procedures for women who use the Act. According to the Lawyers Collective Women’s Rights Initiative (LCWRI), 2013, there have been numerous loopholes in the Act’s implementation. For example, despite the provision of periodic training in the Act for police, health personnel, protection officers, lawyers, and judges, not many states have taken measures for training. Another gap exists in the recruitment of protection officers [42]. Many states have not appointed enough protection officers and many have additional duties [43]. Several states lack a plan for PWDVA implementation and many have not allocated a budget for implementation. There is a lack of periodic or annual aggregated national data from 2006 to 2015 on the appointment of protection officers compared with the actual requirement, training on the PWDVA, or the number of cases filed in each state under the Act [43]. Socialisation of the act needs more attention, particularly with respect to implementation guidelines and monitoring strategy.

Ghosh and Choudhuri (2011) suggest that the promise made by the PWDVA of addressing a case within 60 days from the first date of hearing could not be fulfilled. For many survivors, sluggish implementation of court orders and lengthy legal battles are discouraging. The ambivalent position of the police in upholding survivors’ experience and enforcing judicial orders may have been an impediment to affording women timely and necessary justice. The PWDVA proposes a new emancipatory role for police. However, the perception of police as having power to punish clashes with this role. Police officers can also file cases and take action on their own under the PWDVA. Despite this, the police often do not file a domestic case, but refer the matter to a protection officer [44]. The other barriers to disclosure and help-seeking for IPV are well understood: normalization and minimization, fear of reprisal or escalation of violence, and lack of awareness of or belief in potential support. Many survivors believe that IPV is a burden that must be tolerated so long as it is not perceived as severe, that a husband has the right to use violence against his wife, that violence is the consequence of failings in themselves, or that their partner’s behaviour will change. Often, survivors are reluctant to disclose violence or seek help because of their traumatic experiences. They fear that they will be blamed or not believed. Many justifiably fear for their safety or the safety of their family if they disclose IPV. Some do not disclose to maintain family honour or hope that things can get better. Escalation or reprisal might include increased violence, the onset of physical IPV where it was not already a feature of their abuse, and the loss of their children, home, and financial support. Many survivors have limited awareness of available support services or lack conviction of their effectiveness [2, 12, 19, 20, 45–49]. Legal recourse is often a last resort for survivors generally take because most often, their priorities are met by other mechanisms, such as taking help from informal sources such as family or friends whenever required and violence reduces or stops in her life. However, we did not see an increase in informal help-seeking from NFHS-3 to NFHS-4.

Education, awareness, and mandatory training for the police will provide more effective responses in dealing with cases of domestic violence. As in other countries, primary healthcare providers can and do offer more effective help when they are trained and supported so that the healthcare system is fully engaged to do better. There needs to be a focus on the abilities of police, lawyers, protection officers, and health and social care professionals who are first responders to domestic violence to respond effectively to domestic violence, ensuring the safety of survivors while holding abusers accountable.

To break the cycle of domestic abuse, a concerted community response is essential. The police, lawyers, protection officers, health professionals, social service organizations, and communities must be familiar with national legislation relating to domestic violence. It is the government’s responsibility to prevent and control domestic violence in order to provide its citizens with a life free of violence and characterised by dignity. Local authorities, the health sector, the social services sector, the education sector, the judicial sector, and the law enforcement sector, as well as groups such as women’s unions and the media also need to be involved in raising awareness of the laws intended to prevent and control domestic violence.

### Limitations

The cross-sectional, ecological nature of the data means that we could not establish a causal link between IPV prevalence, help-seeking, and implementation of the PWDVA 2005.

Conclusions and policy implications.

Low disclosure and help-seeking for IPV affect a country’s social, cultural, economic, and political development. An increase in help-seeking could be seen as indicative of the effect of the PWDVA 2005, but use of the act is often a last resort for survivors, who may seek help from family, non-government organisations, and healthcare providers, as well as the police. Unfortunately, we found no evidence of increased help-seeking from either formal or informal sources over the decade after enactment. Whatever the reasons for the disappointing findings, it seems essential to advocate for greater public awareness of the implications of the law. We need to strengthen formal support services so that survivors who seek help receive it and others come to know that they too could be supported. There is a role for community education and mandatory training, at least for the police, to provide more effective responses. Primary healthcare providers can offer more effective help when they are trained and supported, and the healthcare system needs to be fully engaged to do better [50]. We recommend a deeper understanding of help-seeking across all sources to establish a barometer of help-seeking. An increase in formal or informal help-seeking is an indicator of disapproval of violence against women and the enabling environment for tracking progress toward the United Nations Sustainable Development Goals of gender equality and the eradication of all forms of gender-based violence and discrimination.

## Data Availability

Data are available for download via the Demographic and Health Survey (DHS) data distribution system. ICF International owns the DHS website. Visit www.DHSprogram.com for additional information on accessing NFHS data.

## Abbreviations

CI: Confidence interval
IIPS: International Institute for Population Sciences, India
IPC: Indian Penal Code
IPV: Intimate partner violence
LMIC: Low- and- middle income country
NCRB: National Crime Records Bureau, India
NFHS: National Family Health Survey, India
NGO: Non-government organization
OBC: Other backward caste (Indian categorization)
ST/SC: Scheduled tribe or scheduled caste (Indian categorization)
OR: Odds ratio
aOR: Adjusted odds ratio
PWDVA 2005: Protection of Women from Domestic Violence Act, 2005
SDGs: United Nations Sustainable Development Goals
WHO: World Health Organization
